# Bone Health and Physical Activity - The Complex Mechanism

**DOI:** 10.14336/AD.2024.1316

**Published:** 2025-12-17

**Authors:** Alicja Nowak, Małgorzata Ogurkowska

**Affiliations:** ^1^Department of Physiology and Biochemistry, Poznan University of Physical Education, Poznań, Poland.; ^2^Department of Biomechanics, Poznan University of Physical Education, Poznań, Poland.

**Keywords:** physical activity, bone, musculoskeletal system, biomechanical factors, biochemical factors, energy metabolism

## Abstract

This review summarizes the mechanism and role of physical activity in maintaining the proper functioning of the musculoskeletal system. Bone adaptation to the mechanical environment occurs in skeletal regions subjected to the greatest stresses resulting from the nature of exercise, however, there is a varied response of bone tissue to mechanical loads depending on its material and structural properties (trabecular and cortical). The regulation of bone tissue metabolism during physical exercise is influenced by factors associated with mechanical stress (gravitational forces, impact loading, and muscular contractions) as well as by systemic mechanisms (hormones, myokines, cytokines). The presence of insulin receptors and glucose transporters in osteoblasts indicates that these cells consume large amounts of glucose. Therefore, when energy demand during physical activity increases, nutritional factors play an important role in bone response. On the other hand, the musculoskeletal system participates in the regulation of energy metabolism. To maintain bone homeostasis, an optimized form of physical activity should be used (e.g. intensity, duration, training session frequency). The complexity of factors modulating the sensitivity of bones to mechanical stimuli causes the results of physical training are age- and sex-dependent. Moreover, when selecting exercises to improve bone health, it is important to take into account metabolic and musculoskeletal system conditions. In addition, exercise should be safe and adapted to the health and fitness level so as not to increase the risk of fractures. Participation in regular physical activity should continue after the training program to maintain bone mass.

## 1. Introduction

Physical activity is an integral part of a healthy lifestyle, which not only reduces the risk of many diseases, such as cardiovascular diseases, type 2 diabetes, dementia, and cancer, but also helps to maintain normal musculoskeletal function [1-3]. Inactivity, on the other hand, may contribute to health deterioration and predispose to the development or exacerbation of musculoskeletal disorders [4,5]. However, excessive training loads resulting from sports or even recreational activities can contribute to adverse changes in bone tissue [6,7].

The balance between bone formation and resorption is necessary for skeletal health and resistance to mechanical loads and depends on the biomechanical mechanisms [8,9] as well as the action of local factors (growth factors, cytokines, and prostaglandins) [10] and systemic factors, among others calciotropic hormones [11], sex hormones [12-14], growth hormone (GH) [15], and insulin-like growth factor 1 (IGF-1) [16] and also substances released by muscles called myokines [2,17,18]. Moreover, the influence of genetic factors [19] or those related to immune system condition/medications is also important [20,21]. Nutritional factors and energy metabolism also play an important role in this mechanism [22,23].

**Figure 1.**
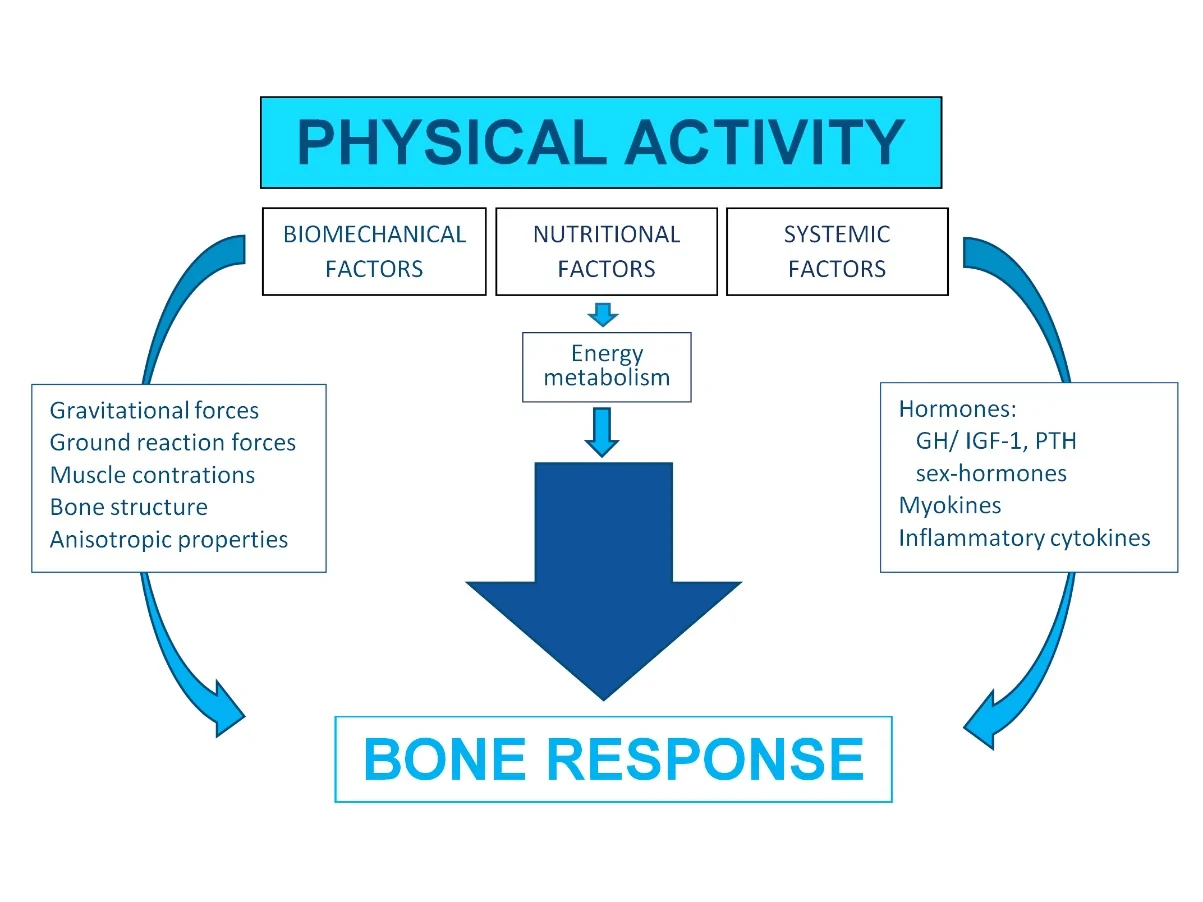
Complexity of the mechanism of the effect of physical activity on bone tissue metabolism.

Taking into account the multi-factor regulation of bone metabolism, the mechanism of bone response to mechanical stress during physical exercise is a very complex process, thereby requiring an interdisciplinary analysis of the problem. This article discusses matters concerning the mechanism of physical activity affecting bone tissue and factors modifying its response to mechanical loads, the knowledge of which may be of importance in the prevention of bone fractures associated with heavy training loads or osteoporotic changes (Fig. 1).

## 2. Biomechanical factors

Physiological bone development during growth and their regeneration in adulthood requires adequate mechanical loads. The response of bone to mechanical stimuli is very complex. Bone has anisotropic mechanical properties, i.e. there occurs heterogeneity depending on the direction of the load generated under physiological conditions. It should also be noted that at the macrostructural level, bones in different parts of the human body and different positions, and different directions in the same position show different mechanical properties [24,25].

### 1.1. Bone response to mechanical stimuli - the structural properties of bone

There is a different response of bone tissue to mechanical loads depending on the properties of its structure: cortical and trabecular. Cortical bone consists of dense and parallel lamellar units and is stiffer than spongy (trabecular) bone which is a highly porous and composed of network of trabecular plates and rods, with less density and a lesser degree of parallel orientation. Trabecular bone is always surrounded by a cortical bone, but different skeletal sites have different ratios of cortical to trabecular bone [24,26,27]. The differences in the structure of the above-mentioned tissues, both in their macro- and micro-architecture, are intended to adapt bone to counteract mechanical loads, including primarily gravitational forces that, depending on the weight of the individual, generate stresses and deformations [25].

The adaptation of bone to mechanical loads occurs in the parts of the skeleton subject to highest stresses resulting from the type of a particular activity, and the shape of the bone is related to its function [9,28]. Osseous trabeculae are "arranged" along the direction of loads, which reinforces the structure. Moreover, the variable arrangement of the trabeculae and the numerous spaces between them throughout the volume of spongy bone cushion the loads, counteracting the structure from becoming too stiff and thus prone to fractures and breaks [25,29]. Consequently, it is found primarily in the epiphyses of long bones, as well as in vertebral bodies.

Rho et al. [28] used ultrasound technology to investigate the mechanical properties of human cortical and cancellous bone described by the longitudinal modulus of elasticity of the femur, tibia, humerus, and lumbar spine, among others. The experimental results showed that there were no major differences in the mechanical properties of the humerus, proximal tibia and lumbar spine. The aforementioned parts of the skeletal system are prepared to absorb greater energies and transform them into deformations of the structure under a given load, which is primarily related to compression forces. It should be emphasised that these are caused by the gravity of the part of the body above the bone in question. Furthermore, as mentioned earlier, bone adapts and remodels in response to the applied load. Consequently, the stiffness and strength of spongy bone in the above bone types were lower than, for example, in the proximal femur [30], in which the resultant force acting on this bone has both compressive components and shear components. Moreover, the angle between the neck and shaft of the femur decreases with age, which may cause an increase in pathogenic shear forces [31]. Hence, this is one of the factors that may cause a decrease in the density of the neck and head of the femur in older people.

It should further be noted that lower stress levels, below the yield point, in the elastic part of spongy bone allow the applied stress to be elastically stored and transmitted, thereby avoiding tissue micro-damage [32, 33]. Conversely, in the plastic part, higher deformation levels, above the yield point, deform the bone material beyond its elastic point, consequently causing damage, usually in the form of microfractures [34]. The strength of spongy bone is clearly related to its ability to store energy elastically and the resulting resistance to micro-damage. Studies by Wirtz et al. [35] have shown that only Young's modulus, or longitudinal elastic modulus (E), and compressive strength are closely correlated with bone density. The mentioned bone features are also related to its porosity [24,36]. Therefore, the E values for spongy bone are considerably lower than for cortical bone, present primarily in the outer layer of bone shafts, including long bones, since the concentric arrangement of bone lamellae and the predominance of one dimension (height) over the other two provides it with greater stiffness compared to trabecular bone. Hart et al. [9] suggests that cortical bone, prior to its damage, can therefore withstand higher stress levels (~150 MPa), but lower strain levels (~2%). The porosity of trabecular bone provides it with greater elasticity compared to cortical bone, enabling it to withstand lower stress levels (~50 MPa), but much greater strain (~50%), before damage. Furthermore, cortical bone is stronger and stiffer when loaded in the vertical direction compared to the transverse or shear direction. Therefore, during physical activity, it mainly participates in providing mechanical support and resists greater forces in the longitudinal direction. Moreover, cortical bone tissue is more resistant to strain at higher velocities and it should have both a high level of stiffness to cope with loads and a high level of strength to resist fractures and deformation-caused damages [34,37,38].

In comparison, the mechanical properties of trabecular bone are less predictable and highly variable, due to the perforated, variable, and less organised arrangement of the lamellae. As mentioned earlier, spongy bone is also present in the vertebral bodies of the human spine, connected to each other via intervertebral discs. Furthermore, the spine should be considered as a biokinematic chain. With the phenomenon of compensation, changes in the structure of one segment affect the entire chain. The clinical manifestation of pathological changes is usually pain caused by pressure on nerve roots. According to the biomechanics of the spine, the vertebral bodies primarily carry axial loads, while the intervertebral discs absorb them. Moreover, stress tests indicate that functional testing of the spine should include evaluation of individual motion segments as a whole [25]. In view of the foregoing, the remodelling of the spongy structure of the vertebrae is related to the phenomenon of pathomechanism of overload changes within the motion segment. Pathogenic loads cause changes in the intervertebral disc [39], which are manifested by its stiffening. Moreover, it loses its cushioning function and the spongy tissue of the vertebral bodies below the altered disc is subject to direct loading. This leads to an increase in the radiological density of the spongy tissue towards values corresponding to those of the cortical bone [40,41]. It should be emphasised, however, that the increased remodelling of spongy tissue in no way resembles the osseous trabeculae of cortical tissue in that, due to its increased density, the spongy bone loses its elastic properties and becomes hard and fragile at the same time. This is very dangerous for high-performance strength and endurance athletes, who experience the above-described pathomechanism of overload, especially of the lumbar spine, often causing compression fractures of vertebral bodies, despite their high density, during competition [40].

A high number of studies on the effect of physical activity on bone tissue is based on measurements of areal BMD. However, other factors, such as bone microarchitecture and geometric properties, determine the fracture risk [26,42]. Also, Rho et al. [28] using computed tomography observed that mechanical properties of bone tissues may result mainly from the different microstructures of each bone, rather than different mineral densities. This fact implies that two individuals with comparable very low bone density values may have different bone strengths. Therefore, the different structure of bones, characterised by a sparse but regular arrangement of osseous trabeculae in relation to each other, as opposed to staggered edges, may result in their greater compressive strength, despite comparable BMD results, towards osteoporosis. Furthermore, although no BMD changes were observed in some studies in postmenopausal women participated in the exercise programs, changes in femur strength index have been reported [43,44]. The strength index of the left hip is automatically calculated during dual-energy X-ray absorbtiometry (DXA) measurements “as the ratio of estimated compressive yield strength of the femoral neck to the expected compressive stress of a fall on the greater trochanter”. It combines the structural parameters with age, weight and height, and may predict hip fracture independently of BMD [42]. Therefore, a comprehensive assessment of the bone tissue response (bone mass together with bone geometric parameters) to the applied intervention could allow a more complete estimation of the effectiveness of the training/exercise program.

### 2.2. Bone response to mechanical stimuli - the specificity of load and type of exercise

The researchers indicate that mechanical stimuli act on bones by inducing stresses (bone deformation), which can be created by gravitational loads, ground reaction forces (GRFs), and muscle contraction [9,17,45,46]. Studies in an animal model have shown that activity-related characteristics of bone loading, such as the direction and magnitude [46, 47] and strain rates [48], the duration of the inter-stimulus interval [49] and the distribution and the number of loading cycles per day [50], are important in the response of bone tissue to mechanical stimulation.

The primary detectors of mechanical stimuli are osteocytes. Osteocyte intracellular network, through gap junctions, connects them to their neighbouring osteocytes and to bone formation cells (osteoblasts) on the bone surface. The extracellular network, through lacunae and canaliculi, extends to the bone marrow, periosteum and blood vessels in the bone [51]. Moriishi and Komori [51] suggested that mechanical stress affects the differential development of the lacunocanalicular structure in the compression and tension sides on the example of femoral cortical bone in wild-type mice.

Mechanical forces induce fluid flow through the lacuna-canalicular system and matrix deformation at the cellular level in bone. In their review, Klein-Nulend et al. [52] suggested that bone strains and loading-induced hydraulic pressure may serve as a mechanical stimulus for osteocytes, and the rate at which the strain is applied is more important than amount of strain. They concluded that given this mechanism, dynamic loads on bone induce an osteogenic response to a greater extent than static loads which was confirmed earlier in animal model studies [53]. Osteocytes are therefore stimulated and transmit biochemical signals which can modulate the recruitment, differentiation, and activity of bone cells [10,52,54].

The previous studies have shown that mechanical stimulation of osteocytes induces release of many signalling molecules, such as adenosine triphosphate (ATP) [55], prostaglandin E2 (PGE2) and nitric oxide (NO) [20], and growth factors such as IGF-1 [56] for bone-forming osteoblasts. Moreover, there was observed that Wnt/β-catenin-signaling pathway is a critical component of the bone response to mechanical stimulation [57,58] and that sclerostin, the SOST gene protein product, a negative regulator of osteoblast activity, is expressed by osteocytes and depressed during mechanical loading [59,60,61,62]. Osteocytes communicate with osteoblasts and bone resorbing cells (osteoclasts) via the sclerostin (SOST)/dickkopf-related protein 1 (Dkk-1)/Wnt axis and also the receptor activator of nuclear factor-κB ligand (RANKL)/osteoprotegerin (OPG) axis. The Wnt/β-catenin pathway plays an important role in osteocyte function and the maintenance of normal bone, whereas Dkk-1 and sclerostin, highly expressed in osteocytes, are negative regulators of the Wnt/β-catenin pathway [59,62,63]. Osteocyte apoptosis decreases the mechanical competence of the bone. This phenomenon may be induced by unloading or by application of excessive strain that induce microdamage within the bone matrix [64].

The classic take on the dependence of bone remodelling on the magnitude of its mechanical stresses is Harold Frost's mechanostat theory. There was suggested that physiological skeletal loading (local bone deformation) enables balanced bone remodelling. Below the lower stress threshold, referred to as the 'Minimum Effective Strains', the stimuli are insufficient to maintain bone remodelling and resorption will be the dominant process, translating into loss of bone mass [65]. This phenomenon occurs in the absence of movement or if mechanical loads are limited, e.g. in astronauts [66,67]. Lack of physical exercise and reduced daily bone mechanical loading leads to increased sclerostin expression in osteocytes, which contributes to inhibition of the Wnt/β-catenin signalling pathway and, consequently, increased bone resorption. In turn, the activation of osteocytes by, among other things, mechanical loads contribute inhibited sclerostin production [59]. Therefore, life under conditions of inactivity or in the microgravity environment of space brings many changes to the human body. The loss of muscle and bone mass are some of the most apparent and potentially detrimental effects of microgravity [66,68].

Osteogenic effects of particular significance are observed in interventions based on high-impact exercise. This type of activity is typical, among others, for jumping training. The effectiveness of this type of exercise is associated with significant bone loading and stress inducing a corresponding osteogenic response. In animal model studies, Judex and Zernicke [48] investigated the results of mechanical stimuli produced by 3-week drop jumping (200 per day, in sets of 50 with brief rest periods between sets, exercise duration 2.5 min/day), on the middiaphyseal tarsometatarsus in growing (12-week-old) White Leghorn roosters. Indexes of bone formation and mechanical parameters were determined in each of twelve 30° sectors subdividing the middiaphyseal cortex. Compared with walking (velocity 0.51 m/s), drop jumping produced a large peak strain rate (+740%) in the presence of moderately increased peak strain magnitudes (130%). Jump training contributed to a significant increase of bone formation rate at periosteal (+40%) and endocortical surfaces (+370%). Another study showed that the interval time between jumping jacks was important in inducing osteogenic effects. Umemura et al. [49], in 5-week-old female Fisher rats subjected to an 8-week training regiment of 20 jumps in one session or in two sessions separated by a 6-hour break, showed that the increase in femur and tibia bone mass, with reference to body mass, was higher compared to the control group. However, the greatest effects were obtained in the group of animals performing jumps in a single session separated by 30-second breaks, compared to the results obtained in animals that had 3-second breaks. This is because longer intervals contribute to an increased sensitivity of bone tissue to mechanical stimuli. Two separated bouts (2 × 10 jumps) were not more effective than a single bout (1 × 20 jumps) daily. The authors suggested that 30-second intervals between jumps might enhance dynamics of the extracellular fluid and could enhance the anabolic effect of loading. Another study by Umemura et al. [69] showed that the effectiveness of jumping training in female Fischer rats (height 40 cm, 100 times/day, 5 days/week for 8 week) for bone hypertrophy (fat-free dry weights of the femur and the tibia) was not limited by age.

Studies in humans also indicate significant effects of jumping-based exercise on changes in bone mass and strength. A study in girls aged 9 to 12 years who undertook exercise involving jumps (during intervention increased the number of jumps from 10 to 20 and the height from 10 to 50 cm) three times a week for seven months showed a significantly greater increase in BMD at the femoral neck (+2.6%) and intertrochanter (+1.7%) and also the structural changes (increased bone cross-sectional area and section modulus at the femoral neck, which translated to significant gains in bone strength) in early-pubertal (but not prepubertal ) girls compared with the control group [70]. Kato et al. [71] conducted a study in adult young women (mean age about 20 year) who performed only 10 maximum vertical jumps with both feet for 6 months, 3 times a week. The interval between the jumps was about 8-12 seconds. The session therefore lasted less than 2 minutes. After the exercise program, BMD of the femoral neck and lumbar spine increased significantly in the jump women compared to baseline, but there was no significant change in the control group. Therefore, a number of repetitions as high as in the previously discussed study in animals is not required to induce osteogenic effects. Kato et al. [71] suggested that in jump training, not only the ground reaction forces occurring during landing after a jump, but also the muscle contraction forces acting on specific bone sites may play a role in bone adaptation.

During physical activity, contracting muscles create mechanical stresses on the bones, hence some studies have emphasised the dependence of bone mass on muscle strength, in particular during growth. In the literature, this phenomenon is referred to as the muscle-bone unit [72]. The effect of skeletal muscle on bone tissue has a biomechanical [46] as well as biochemical dimension because is related to the production of molecules called myokines by muscles during movement - “secretory crosstalk” [2,18], which will be discussed later in this article. Therefore, in human studies, resistance training has often been successful in inducing significant changes in muscle [73] and bone mass [74,75]. In a meta-analysis involving 19 studies (919 subjects), Wang et al. [74] examined the results of different intensities and frequencies of resistance training on lumbar spine, femoral neck, total hip and trochanter BMD in postmenopausal women. The authors showed that moderate intensity resistance training (65-80% 1 repetition maximum) for 3 days a week (superior to 2 days/week) was relatively effective in improving bone mass of the mentioned skeletal fragments. They noticed that especially changes in the femoral neck and lumbar spine BMD were much significant. However, the results of the resistant training depend on the intensity. In a single-blind, randomized study, performed in postmenopausal women with the hip and/or spine T-score < -1.0, Watson et al. [75] observed that 8-month of supervised high-intensity resistance training (5 sets of 5 repetitions, >85% 1 repetition maximum) program, performed twice a week for 30 minutes, induced significantly greater improvements in bone mass and femoral neck structure and physical function than home-based a low-intensity exercise program (10 to 15 repetitions at <60% 1 repetition maximum).

The importance of muscle work in inducing the response of bone tissue to mechanical loading has been particularly studied by Matijevich et al. [76]. In a study on ten healthy subjects, they confirmed that lower leg skeletal muscle strength loads the tibia more during running than ground reaction forces (GRF). Therefore, it should not be assumed that an increase in GRF is the only indicator of an increase in load on the tibia or risk of overload injury during running. Furthermore, Matijevich et al. [76] suggested that during running, the peak GRF of the tibia are typically 2 to 3 times the body weight, whereas the peak forces acting on the distal end of this bone are typically 6 to 14 times the body weight. It should be noted that the loading of lower leg bones results primarily from the muscles responsible for movement and stabilisation of the knee and ankle joints. Moreover, it occurs when active and passive stabilisers are overloaded. According to the pathomechanism of fatigue fractures, it should be emphasised that it begins with fatigue of muscles, which are unable to perform the cushioning role, followed by transfer of loads onto passive stabilisers, then joint structures are overloaded resulting in all the loads being taken over by bone tissue. As a result of, for example, training spongy bone located in the epiphysis of the proximal tibia undergoes microdamage and remodelling to increase its density, which reduces its compressive strength [29,40].

Moreover, the force developed by the muscle acts on the mobile part of the joint, through the tendon attached, in this case, to the tibial shaft. However, as mentioned above, the shaft of the long bone is made up of cortical bone tissue, which has very good stiffness and strength properties, but in relation to vertical loads, and much less in relation to transverse or shear loads. Therefore, a fatigue fracture of the tibia may occur, but it will not be a compression fracture and an increase in loading forces acting on the tibia may occur without an increase in GRF. Furthermore, assuming a standing position with flat feet compared to standing on one’s toes results in the same GRF, but in the latter case the bone loading force may be significantly higher due to the lower leg muscle force [76].

## 2. Systemic mechanisms and energy metabolic factors

In addition to mechanical factors, hormonal, metabolic or nutritional factors also play an important role in modifying the sensitivity of bone tissue to mechanical loads associated with physical activity. Both a single exercise stimulus and systematic training, depending on the level of fitness and metabolic conditions, significantly determine the homeostasis of the organism and can thus influence the adaptation of bone tissue to exercise [77,78]. Moreover, during inflammatory diseases, systemic cytokines might also affect bone mass and reduce the response of osteocytes to mechanical loading. In a study on MLO-Y4 mouse osteocytes, Bakker et al. [20] found that proinflammatory cytokines, both tumor necrosis factor α (TNF-α) and interleukin 1β (IL-1β), inhibit the up-regulation of NO production in osteocyte after mechanical stimulation by pulsatile fluid flow. They also observed that incubation with IL-1β for 24-hours stimulates osteocyte apoptosis.

### 2.2. Hormones and sensitivity of bone to mechanical stimuli

Hormones significantly affect the sensitivity of bone tissue to mechanical stimuli are the GH, sex hormones, and PTH [11-16]. Among other things, these hormones affect osteocyte function. Osteocytes, which play a pivotal role in bone homeostasis, integrate hormonal and mechanical stimuli in regulating bone remodelling [79,80].

Studies in an animal model have documented that GH increases the sensitivity of bone tissue to mechanical stimuli, reducing the threshold for bone adaptation to mechanical loads [15]. Thus, the significant sensitivity of bone to mechanical loads during growth is primarily related to the aforementioned hormone. The anabolic action of the GH/IGF-1 axis in bone is important for longitudinal growth as well as for obtaining adequate bone mass during adolescence and early adulthood. Serum GH levels decrease with age, with daily GH secretion in older men constituting 1/5 to 1/20 of that in young adults [81]. In the study performed in young and aged mice, Liu et al. [79] showed the effects of GH receptor (GHR) ablation on mitochondrial function in cortical bone osteocytes. Using in vivo multiphoton microscopy method, they demonstrated reduced mitochondrial volumetric density and reduction (>10%) in mitochondrial membrane potential in GHR null osteocytes. The authors suggested that ablation of GHR in osteocytes, in an age- and sex-dependent manner, makes these cells vulnerable to metabolic disorders. They concluded that their data were consistent with previous studies which showed the protective effects of GH and IGF-1 on mitochondrial condition with regard to reactive oxygen species (ROS) metabolism and that deterioration of GH signalling due to ageing impares mitochondrial function in osteocytes, causing changes in bone strength.

Other hormones that sensitises bone tissue to mechanical stimuli are sex steroids [12,80,82]. Sex steroid deficiency is associated with bone fragility and bone loss, including through osteocyte apoptosis [83]. Nelson et al. [82] suggested that menopause significantly affects bone response to plyometric exercise in women. The authors examined the levels of bone turnover markers, such as procollagen type I amino-terminal propeptide (PINP) and c-terminal crosslinking telopeptides of type I collagen (CTX-I) and, as well as sclerostin and Dkk-1, at rest and after (5 minutes, 1 hour, 24 hours postexercise) a single series of plyometric exercise (128 jumps, organized into 5 circuit stations) in 20 premenopausal and 20 postmenopausal women. They noticed anabolic effects only in younger women. In a cross-sectional study, Khosla et al. [14] investigated the relationship between volumetric BMD (vBMD), size, geometry, and structure at different skeletal sites (using quantitative computed tomography) and serum bioavailable estradiol and testosterone levels in age-stratified women. They found that none of the vBMD/structural parameters were associated with bioavailable estradiol levels in young premenopausal women (aged 20-39 yr), who were estrogen repleted, but in elderly postmenopausal women (aged 60 yr and older) they showed highly significant associations between trabecular and cortical vBMD parameters with this hormone levels. Moreover, they documented that trabecular bone is more responsive than cortical bone to decreasing serum concentrations of this hormone.

The effect of sex steroids on bone tissue is exerted through estrogen and androgen receptors (ERα, ERβ, AR), which were detected in bone cells [12]. Despite the decline in estrogen levels in women and testosterone levels in men, which is the beginning of rapid bone loss, estrogen is also important in regulating bone metabolism in men, and serum levels of bioavailable estradiol gradually decline with age in men [13]. In a study of osteocyte estrogen receptors (ERβ), Xu et al. [80] determined their role in bone morphology and skeletal response to mechanical loading in young and adult male and female mice. The authors revealed the role of ERβ in osteocyte-regulated bone turnover in a gender-dependent manner. They observed that young male mice with ERβ osteocyte deletion, relative to control littermates, had upregulated expression of androgen receptors while this was not noted in female mice, suggesting a compensatory effect of testosterone on male bone protection.

In an animal model study, it was observed that estrogen in osteocytes inhibits the expression of sclerostin - the product of the SOST gene. Sclerostin and (Dkk-1) are secreted by osteocytes and play pivotal roles in the pathogenesis of post-menopausal osteoporosis [84]. Dkk-1 and sclerostin inhibit bone formation by blocking the osteoblast Wnt/β-catenin signalling pathway. Wnt/β-catenin signalling pathway plays a significant role in controlling bone formation and bone resorption, which makes it an important determinant of bone mass [85]. In a study conducted on human cells, Kim et al. [86] showed that sclerostin gen (SOST) expression is negatively regulated by estrogen signaling in osteoblasts through interaction with bone morphogenetic protein 2 signaling and involving the Wnt/ERα and β-catenin pathways. Armstrong et al. [58] showed that in the absence of ERα (the study on the mice osteoblastic ROS 17/2.8 cell line and in primary cultures of osteoblast-like cells), the Wnt pathway contribution to bone cells adaptive response to strain was severely limited.

Several findings suggested that PTH also has a potential role in bone anabolism during mechanical loading. In the animal study, Tirado-Cabrera et al. [87] indicated that the presence of both functional primary cilium and PTH receptors (PTH1R) in mechanically stimulated osteocytes is essential for proper communication with osteoclasts and reduces osteoclastic cell formation, including by regulating IL-6 secretion. Moreover, this hormone regulates the expression of sclerostin. Bellido et al. [11] showed that continuous excess of PTH in mice dramatically decreases the SOST/sclerostin expression in osteocytes and this mechanism proposed for hormonal control of osteoblastogenesis. The authors suggested that intermittent injections of PTH only transiently affected SOST mRNA levels.

### 2.3. The role of myokines in bone metabolism

During physical activity, molecules secreted from muscles called myokines play an important role in bone metabolism. These molecules are involved in the regulation of processes undergoing in skeletal muscles i.e. muscle remodelling, repair, and maintenance and affect many organs and tissues, including bone tissue (muscle-bone crosstalk) [1,2,18]. Sui et al. [18] classified myokines such as IGF-1, fibroblast growth factor 2 (FGF-2), irisin, secreted acid- and cysteine-rich protein (SPARC), matrix metalloproteinase 2 (MMP-2), bone morphogenetic protein 1 (BMP-1), brain-derived neurotrophic factor (BDNF) and β-aminoisobutyric acid (BAIBA) into the category of bone-forming factors. However, in the case of low physical activity or immobilisation, increased secretion of myostatin by skeletal muscles negatively regulates myogenesis [88]. Qin et al. [85] suggested that myostatin may have an indirect inhibitory impact on osteoblast activity and bone formation through products released by osteocytes. In the study performed on mouse cultured osteocytic (Ocy454) cells, they demonstrated that the treatment of osteocytes with myostatin leads to significant increases in the expression of sclerostin and Dkk-1 (two Wnt signalling inhibitors). The authors have also shown that myostatin significantly increases the expression of RANKL in osteocytes, thus myostatin may indirectly promote osteoclast differentiation, activity, and viability, presumably associated with enhanced bone resorption.

In the human study, Kim et al. [89] demonstrated that resistance loading (3 sets of 8-12 repetitions to volitional fatigue of squat, leg press, knee extension) downregulates myostatin expression and alters genes which are key to cell cycle progression in adult subjects. They also assessed influences of age and gender on key transcriptional activities after loading and observed that young men (20-35 yr) reacted most positively to the loading program, demonstrating significant changes in myostatin (inhibition) and factors thought to promote the growth and regeneration (among others load-sensitive mitogen mechano-growth factor MGF). The older women (60-75 yr) were the least responsive group.

Irisin is a myokine secreted mainly by muscle during physical activity and distributed through the blood [90]. However, other tissues, including adipose tissue, are also sources of irisin [91]. Associations between circulating irisin levels and insulin resistance may indicate irisin role in energy processes, especially glucose metabolism. In a cross-sectional study involving 1115 obese Chinese adults, Shi et al. [92] found that circulating irisin levels were significantly decreased in subjects with insulin resistance than those without, and elevated circulating irisin were independently associated with reduced risk of insulin resistance after adjusting for potential confounders. In the animal and human study, Zügel et al. [91] showed a sexual dimorphism for the irisin at rest and in response to exercise in normal-weight but not in obese subjects. They observed that transient increase in serum irisin levels after acute exercise (a step-wise incremental exercise trial until exhaustion on a cycling ergometer) was stronger in lean women compared to men, supporting findings of a positive association between estradiol and irisin.

Irisin plays an important role in the regulation of bone homeostasis. Tsourdi et al. [3], in their review, summarized data on the role of irisin in bone homeostasis and included 37 abstracts on the irisin and bone status in healthy subjects and suffering from primary or secondary osteoporosis. They concluded that most human studies have showed positive correlations, while others have suggested no correlations between irisin and BMD results. The authors stated that recent findings indicate the need for improved methods to measure circulating irisin levels. In their animal study, Colaianni et al. [93] demonstrated that recombinant irisin (r-irisin), when injected into mice, increased cortical bone mass and altered bone geometry typified by an increase in periosteal perimeter. The authors observed that the effects of irisin on osteoblastic bone formation were mainly caused by the suppression of sclerostin. Ning et al. [94] suggested that irisin regulates bone metabolism in part by binding it to integrin proteins to activate signalling pathways and this myokine plays an important role in chondrocyte homeostasis. In multiple experiments with primary murine progenitors and the RAW 264.7 macrophage cell line, Estell et al. [95] demonstrated that irisin plays an important role in stimulating osteoblasts and osteocytes, but also directly increases osteoclasts differentiation and promotes bone resorption. Therefore, it may also be an important counter-regulatory hormone.

Irisin inhibits bone loss under pathological conditions and during exercise affects bone metabolism by regulating muscle and bone cells. In the study conducted on mice exposed to hind-limb unloading, Colaianni et al. [96] showed that r-irisin treatment mitigates bone loss and muscle atrophy and postulated that an irisin-based therapy may be a strategy for the prevention and treatment of both osteoporosis and sarcopenia in patients during reduced mobility and in astronauts exposed to microgravity. In the study which used hind-limb unloading mice model and a random position machine to simulate microgravity in vivo and in vitro, Chen et al. [97] demonstrated decreased bone formation and osteoblast differentiation, and downregulated expression of fibronectin type III domain-containing 5 (Fdnc5; irisin precursor). However, they observed that treatment with recombinant irisin (r-irisin) positively regulates osteoblast differentiation under simulated microgravity through increasing β-catenin expression.

Boström et al. [90] examined irisin blood levels after exercise in mice and healthy adult humans. They observed that plasma concentrations of irisin in mice were elevated by 65% after three weeks of free wheel running; in human subjects this protein increased 2-fold after 10-week supervised endurance exercise training, compared to the non-exercised state. The increase in circulating irisin in both species was roughly proportional to the increases observed at the mRNA level in muscle. In their review, Ning et al. [94] stated that numerous studies showed the release of irisin after exercise, however, the results on irisin expression in different types of exercise are inconsistent. The changes in its levels were related to the intensity and form of the exercise, as well as the physical condition and metabolic level of the subjects.

### 3.3. Nutritional factors and energy metabolism in bone response to physical activity

Nutritional factors play an important role in maintaining bone tissue homeostasis, which is of particular importance in people with increased physical activity [22,23]. In a meta-analysis of studies in children and adolescents (aged 3 to 18 years), Specker et al. [98] observed that the level of calcium intake had a significant effect on the response of bone tissue to exercise (increase in leg bone mineral content, BMC). Matias et al. [99] found that magnesium intake was a significant predictor of total BMD in young elite swimmers. However, Fouhy et al. [100] documented the importance of dietary calcium to magnesium (Ca:Mg) ratio for bone health. They observed that the Ca:Mg ratio at the middle tertile (2.2-3.2), in comparison to the highest or lowest Ca:Mg tertiles, was associated with higher bone mass at four anatomical sites (lumbar spine, femoral neck, trochanter and total hip BMD) and with lower risk of osteoporosis. The authors suggested that the suboptimal magnesium status is associated with low calcium absorption due to reduced vitamin D and PTH activation.

In addition to providing adequate amount of calcium and magnesium, for optimal bone health are also required elements such as zinc [101], iron, as well as vitamins D [22], K, C, E, A, and B [102]. Amino acids are essential for collagen synthesis and hydroxyapatite mineralization in bone [103]. Iron is also involved in collagen production but also participates in metabolism of vitamin D [104].

The primary role of vitamin D is to maintain calcium homeostasis and intestinal calcium absorption. In a study on male subjects (76 athletes and 37 non-athletes, aged 15-19), Malczewska-Lenczowska et al. [22] investigated the impact of vitamin D (the serum level of 25(OH)D total and free fraction) and iron status (i.e., serum ferritin or soluble transferrin receptor), and also calcium intake (ADOS-Ca questionnaire) on lumbar cortical and trabecular bone. The obtained results showed the positive relationship of vitamin D levels and iron status with cortical, but not trabecular, bone health but only in physically active subjects.

The vitamin D status is also important for skeletal muscle condition. In a study conducted on male sweep-oar rowers and canoeists, the relationship between serum levels of 25(OH)D, measured in autumn, and biomechanical parameters (peak torque of muscles involved in the rowing cycle) confirmed the role of vitamin D in maintaining skeletal muscle health [105]. In a study conducted on a large group of athletes, Krzywanski et al. [106] documented that at different times of the year, 25(OH)D concentrations in athletes training outdoors were significantly higher than in indoor athletes. Therefore, in physically active people, outdoor activities can be important in maintaining 25(OH)D levels, even outside the summer season.

The fact that free radicals are generated during exercise, the intake of vitamins with antioxidant properties may be important. Hagan et al. [107] suggested that vitamin E may impact osteocyte survival and bone adaptation to loading because of its antioxidant properties. In an animal model, they showed that depleting vitamin E in the diet (for up to 11 weeks) caused increased oxidative stress in osteocytes and impaired their survival after the intervention of 5 weeks of daily treadmill exercise. However, Stunes et al. [108] suggested that care should be taken before recommending antioxidants supplementation to subjects with normal circulating concentrations of these vitamins. In a double-blinded randomized placebo-controlled experiment, they showed that supplementation with high doses of vitamin C (1000 mg/day) and vitamin E (235 mg/day) reduced increase in aBMD in elderly, healthy men after 12-week supervised strength training (3 sessions/week and 3-15 repetitions maximum sets/exercise). The observed increase in aBMD in the control group was accompanied by reduced sclerostin concentrations and elevated concentrations of IGF-1 and leptin, beneficial for bone formation.

Bone metabolism - bone remodelling activity - requires an adequate supply of energy, the demand for which increases during exercise. The presence of insulin receptors and glucose transporters in osteoblasts involved in insulin-independent (in immature osteoblasts GLUT-1) and insulin-dependent (in mature osteoblasts GLUT-4) glucose transport into these cells indicates that these cells consume a large amount of glucose, which is an energy substrate for them [23,109,110]. Glucose is necessary for osteoblast differentiation and the synthesis of type I collagen, the main component of the bone matrix [110]. Moreover, Liu et al. [79] suggested that osteocytes are very sensitive to glucose deprivation in vitro and they found that glucose deprivation (4 to 6 hours) induced osteocyte apoptosis in mice.

Heinonen et al. [111] measured femoral bone blood flow and glucose uptake at rest and during exercise in young healthy subjects using positron emission tomography. They indicated that one-leg intermittent isometric exercise increased femoral bone blood flow from rest to low intensity exercise and dynamic one-leg exercise increased femoral bone glucose uptake almost fivefold compared to the resting leg, suggesting that metabolic requirements of bone during the movement are enhanced.

The mutual association between carbohydrate and bone metabolism has been confirmed and it was demonstrated the contribution of uncarboxylated osteocalcin (OC), an osteoblast derived osteokine, released from bone, in β-cell proliferation [112]. Proper glucose uptake by osteoblasts is necessary for the expression of the hormone osteocalcin and the regulation of carbohydrate homeostasis [110].

The supply of energy substrates in physical exercise affects the rate of secretion of interleukin 6 (IL-6) from muscle tissue [113,114]. The role of this muscle-derived pleiotropic cytokine is to maintain homeostasis both locally (within the muscle) and systemically (released into the circulation) by increasing the availability of energy substrates. The levels of IL-6 may increase up to 100-fold in response to exercise and decline in the period following exercise, and its expression, among others, is upregulated in response to low muscle glycogen [108]. Dhamrait et al. [115] demonstrated an association between a functional polymorphism in the IL-6 gene and femoral cortical remodelling during strenuous physical exercise in male army recruits and suggested a fundamental role for IL-6 in driving bone resorption. Nutritional interventions may affect the IL-6 response to exercise in healthy subjects [114]. In their study, Sale et al. [116] observed the metabolic response of bone to carbohydrate supply during treadmill running in healthy, physically active men. In a trial during which subjects consumed carbohydrates, there was a significantly smaller increase of bone resorption marker (CTX) and IL-6 in blood for several hours after exercise (120 minutes of treadmill running at 70% of maximal oxygen consumption), compared to a placebo test. The authors of these studies concluded that carbohydrate supply during exercise, applied immediately before exercise, every 20 min during and immediately after exercise (0.7 g carbohydrate per kg body mass per hour), may be beneficial for the skeletal system [116]. This is particularly important for endurance athletes, whose long training sessions significantly increase energy requirements. In some studies, conducted in people practising such sports (long-distance running, professional cycling), an adverse effect of prolonged training (length of distance covered) on bone metabolism was observed [6,7]. Pollock et al. [7] determined bone mass in elite female endurance runners and noted low BMD in 34.2% of the athletes at the lumbar spine, and osteoporosis in 33% at the radius. Moreover, in longitudinal analysis a positive association between training volume and the BMD reduction at the lumbar spine was identified. Olmedillas et al. [6] observed the lower BMC and BMD (in regions of hip, pelvis and femoral neck) in adolescent cyclists in comparison to healthy age-matched controls. Ihle et al. [78] observed the dose-response relationship between energy availability and selected markers of bone turnover in regularly menstruating, sedentary young women of normal body composition. They suggested that militaryservice women and others involved in physical training programs may need to maintain their energy availability above 30 kcal/kg lean body mass/day to avoid increased bone resorption.

### 3.4. Systemic factors and bone stress injuries

Fredericson et al. [77] suggested that up to 20% of collegiate endurance runners sustain one or more bone stress injuries per year. The increased risk of fatigue fractures in individuals engaged in prolonged physical activity (runners, military personnel) is thought to be primarily related to biomechanical factors, as well as energy deficit and associated hormonal dysfunction, i.e. the so-called female athlete triad [77,117,118]. Fredericson et al. [77] evaluated the effect of nutrition education on bone stress injuries in female distance runners and found that a nutritional intervention that optimises energy availability may reduce trabecular-rich bone stress injuries. In addition, in the study of military recruits, Ruohola et al. [119] showed that a lower serum concentration of the vitamin D metabolite (25(OH)D) may be a predisposing factor for stress fractures. In a prospective cohort study of 6712 girls (aged 9 to 15 years) who engage in high levels of high-impact activity (at least 1 hour/day of high-impact activity: basketball, running, soccer, tennis, cheerleading, or volleyball), Sonneville et al. [120] observed that vitamin D intake (both dietary and supplement intake) was predictive of a lower risk of developing a stress fracture.

Another negative factor of bone response to exercise may be a disruption of Ca homeostasis and increased PTH secretion. Kohrt et al. [121] suggested that rate of bone resorption, as measured by serum CTX concentration, increases after 60 minutes of intense cycling exercise to mobilize Ca from bone and defend against the decrease in serum ionized Ca concentration. Milk is rich in electrolytes, including Ca, and it is considered for use during or after exercise to decrease metabolic disturbances. A study performed by Prowting et al. [122] in untrained healthy adult females (age about 20 y) showed that drinking 550 ml of milk at 5 minutes and 1 hour post-exercise (combined plyometric and resistance exercise, ~75% 1-RM) resulted in a smaller increase in the bone resorption marker (CTX), as measured by area under the curve analysis from 75 minutes to 48 hours, compared with consumption of a carbohydrate drink (52.7 g maltodextrin in 550 ml water) [122]. In the study performed in young women and men, Shirreffs et al. [123] showed that milk (compared to the same volume of water or sports drink) is a potential candidate for an effective post-exercise rehydration, because of its high electrolyte content and the presence of carbohydrates in a concentration similar to many commercially available sports drinks (except individuals with lactose intolerance).

## 4. Age-related differences in bone tissue response to physical activity

As the sensitivity of bone tissue to mechanical loads is the result of a complex interaction of biomechanical factors, including those dependent on the structural properties of bone, as well as systemic factors, its response to physical activity varies during ontogeny period.

During adolescence, particularly in the pre-pubertal period, when bone tissue is most sensitive to mechanical loads, physical activity can cause a significant increase in the rate of bone mass gain and changes in bone architecture [98,124,125]. Although genetic factors are known to have a significant effect on bone mass gain [126], physical activity undertaken during this period can significantly improve the condition of this tissue [130,131].

Studies conducted by some authors have shown that physically active children and adolescents, e.g. those involved in sports training or recreational activities, usually have higher bone mass compared to the control group and usually have a greater increase in bone mass than less active children, but not all types of physical activity have such unequivocal effects [98,125,127]. Bailey et al. [127] in a study conducted over a 6-year follow-up period in children aged 8 to 14 years found that size-adjusted BMC of the whole skeleton (one year after reaching peak gain rate) was greater in active boys and girls by 9% and 17%, respectively, compared to their inactive peers. Specker et al. [98], on the basis of a meta-analysis of 22 trials, concluded that bone-loading exercise interventions (mainly resistance and high-impact activities, duration of the intervention from 3 to 36 months) in children and adolescents aged 3 to 18 years, compared to the control group, lead to 0.6% to 1.7% greater annual increase in bone accrual. However, the greatest magnitude of change in bone mass was mainly in pre-pubertal children (no sex differences in the response to exercise); the authors did not observe any such significant and unequivocal effects in older children (early or post-pubertal).

The relationship between the magnitude of bone tissue changes and the stage of ontogeny at which training began was also confirmed in the study by Kannus et al. [128] in female tennis and squash players which assessed the differences in BMC between the dominant and non-dominant arm. The differences in these parameters were significantly greater in women who started training at or before menarche, compared to women who started training after this period. However, skeletal benefits induced by systematic training during growth may be maintained in the long term [129], for example, higher bone mass was observed in master athletes in comparison to non-active controls [130,131].

Despite the significant effect of resistance or jumping exercises on the growth of bone mass in children, the safety of performing such exercises over a longer period of time should be considered. Faigenbaum and Myer [132] point to the risk of growth plate damage, especially when young athletes perform jumps that involve ground reaction forces of five to seven times their body weight. They point out that the growth plate can be much weaker than the surrounding connective tissue and less resistant to shear and tensile forces. Damage to this part of the bone can cause growth impairment, as can excessively loading during resistance exercise. Sports disciplines which involve exercises with age-appropriate loads can contribute to a very early onset of problems with the musculoskeletal system in young athletes. For example, studies carried out in young adult rowers and field hockey players (who started their first training during childhood or adolescence), have shown a picture of complete overload lesions at different levels of the intervertebral discs of the L5-S1 spine [29,40]. As described above, changes in intervertebral discs, manifested primarily by their stiffening, affect the spongy tissue filling the vertebral bodies towards increasing their density, thereby decreasing their elasticity. Bone becomes brittle, so a process can begin in a young athlete which may lead to compression bone fracture in the future. Therefore, in order to minimise the occurrence of the above problem among athletes, it is necessary to carry out selection of people wishing to practice particular sports professionally consisting in verification of the correct functioning of their musculoskeletal system, in particular the spine. Periodic examinations in the form of functional tests for detailed diagnosis of the musculoskeletal system are also necessary. It should also be proposed that appropriate specialists be trained to carry out such selection and control examinations of athletes who wish to practise or already practise various sports, and that the control of exercise be entrusted to competent physiotherapists.

During adulthood and ageing, the sensitivity of bone tissue to mechanical stimuli decreases and in women it is particularly impaired during menopause and post-menopausal period. Changes in hormonal status with age, mainly estrogen [13] and GH [81], are an important factor in reducing the sensitivity of bone tissue to mechanical stimuli. Using reconstructed confocal images of bone with fluorescently labeled mouce osteocytes, Schurman et al. [133] showed significant changes in the lacuno-canalicular network during aging which may cause changes in osteocyte mechanosensitivity. Moreover, Holguin et al. [134] assessed histomorphometry of young, adult and old C57Bl/6JN mice subjected to 5 days of tibial compression and observed that bone formation response of aged mice to loading was reduced due to impaired Wnt activity [134]. Ageing is also associated with accumulation of adipocytes in bone marrow cavities, which contributes to the impairment of bone tissue regeneration [135] and also with changes in the production and activity of inflammatory cytokines, chemokines, and growth factors, which lead to dysregulation of the bone-immune axis [136]. Structural and functional changes in the muscle system also occur during ageing [137]. The potential for muscle acting on bone tissue decreases due to changes in muscle tissue, such as decline in muscle size and function [137], and myokine levels decrease [138]. As a result, the mechanical and biochemical effects of muscle on bone tissue are compromised.

Despite the reduced sensitivity of bone tissue to mechanical stimuli, physical activity undertaken during adulthood and ageing is important for maintaining healthy bones, which plays a significant role in the prevention of osteoporosis-related bone fractures. Increased bone mass or decreased bone degradation in certain skeletal segments has been reported following targeted training programs or in physically active subjects compared to less active people, however studies show mixed results [139-142]. Rodríguez-Gómez et al. [5] determined the relationship between bone mass and movement behaviours in 871 older people and observed that the combined effects of physical activity were significantly associated with leg BMC and BMD, femoral neck BMC and whole-body BMD. These associations were gender specific because daily movement behaviour was associated with leg and pelvic bone mass in the men’s subgroup and, whole body, leg and arm bone mass in women.

Regarding the type of physical activity, a systematic review and meta-analysis by Oliveira et al. [143] presented evidence that exercise and sports enabling weight-bearing practice are effective to increase bone mass. The osteogenic stimulus was effective in the bone sites of the lower limbs, hip, and spine. Therefore, for the enhancement of bone health the authors recommended exercise and sport practice, in which appropriate control of training loads is ensured. Sanchez-Trigo et al. [144] conducted a systematic review and meta-analysis of prospective randomised controlled trials, comparing at least one exercise group vs. a control group with sedentary lifestyle or sham exercises. In this study, the authors assessed the effect of non-supervised physical activity intervention (from six months to two years) on the femoral neck and lumbar spine BMD in adult women (aged ≥ 30 years). The authors found that primarily interventions featuring training with dynamic skeletal loads (so-called weight-bearing and impact exercises), e.g. jogging, jumping, running, dancing, and vibration platform training, resulted in BMD changes in the femoral neck area. The effects of the intervention in terms of femoral neck and lumbar spine BMD were more pronounced in women with osteopenia or osteoporosis, compared to healthy women. The results of the studies indicate that the magnitude of the effect of physical activity on bone tissue in adult women is primarily determined by the type of exercise used and bone mass status.

Moreover, in the elderly, exercises may improve the muscular system and prevent fall-related fractures, especially in people with low bone mass [141,145,146]. In a meta-analysis covering nine studies, Moran et al. [147] showed that jump training can be effective and safe in increasing muscular power in older non-obese adults. The authors suggested that more than three jumps per set (up to ten jumps), and two to three sets per exercise (60 seconds of recovery between sets), repeated up to three times a week, could be beneficial. However, they suggested that practitioners must consider some individually specific factors when formulating jumping training programs. Clemson et al. [145] introduced the Lifestyle integrated Functional Exercise (LiFE) program for fall prevention. The authors suggested that balance enhancing activity and lower limb strength training are the optimum modality for fall prevention in older adults. They tested the LiFE program, which incorporates such exercises into habitual daily routines. It resulted in a 31% reduction in the rate of falls compared with the control program, which involved gentle sham exercise.

Although according to studies resistance exercise as well as impact exercise were effective and the most recommended in relation to bone mass and fall prevention in adults and in the elderly, participation in exercise by less fit and older people may increase their risk of bone fractures. Watson et al. [75] monitored adverse events of an 8-month, twice-weekly, 30-minute, high-intensity resistance and impact training (5 sets of 5 repetitions, >85% 1 repetition maximum) program for post-menopausal women with low to very low bone mass and showed that a brief exercise intervention induced no adverse events under highly supervised conditions. Exercises were efficacious for enhancing femoral trabecular and cortical vBMD, as well as functional performance. An additional aspect to consider in the selection of resistance exercises is body weight and musculoskeletal overload changes resulting from work, even in sitting position.

The most effective exercises for the prevention of osteoporosis are those that significantly load the skeleton but endurance exercises as walking or cycling have limited osteogenic effects [148,149]. However, it should be emphasized the effectiveness of these forms of physical activity in the prevention of metabolic disorders [150,151], which may also be important for bone health. Indeed, it has been demonstrated that aging and obesity may induce adipocyte accumulation in bone marrow cavities, which can exacerbate inflammation and contribute to bone health deterioration [152,153]. Therefore, it can be assumed that physical activity may improve bone quality independently of mechanical stimulation but also as a result of its favourable metabolic changes in adipose tissue. Fonseca et al. [154] showed that ovariectomy in Wistar rats lead to an increase in visceral and bone marrow adipose tissue. The authors documented associations of bone marrow adiposity with, among other things, bone resorption rate (CTX), higher empty osteocyte lacunae, number of oxidatively damaged osteocytes and inverse relationship with biomechanical properties of the femur (deterioration in cortical geometry, trabecular microarchitecture and resistance to fracture). They observed that the amount of aerobic physical activity (assessed by the running distance on the wheel) inversely correlated with visceral and bone marrow adiposity. Therefore, it seems that programs using endurance-type activities may be recommended during ageing (especially in the postmenopausal period) or for people with visceral adiposity to improve bone health. This issue requires further research, as in human studies such forms of activity have also had negative effects on bone tissue, especially during weight reduction [155]. Several authors proposed to incorporate impact activities in endurance training programs [149]. Armamento-Villareal et al. [155] suggested that both resistance and combined aerobic and resistance exercise can be recommended to protect against bone loss during weight loss therapy of older adults with obesity. Based on their findings, Colleluori et al. [156] observed that combined aerobic and resistance exercise is superior to either mode independently for maintaining muscle mass during weight-loss therapy.

**Table 1:** The complexity of the mechanism of bone response / adaptation to physical activity.

Factors determining bone response/adaptation to physical activity	Examples of the mechanism of bone response/adaptation to physical activity	Reference
**nature of bone load during physical activity**	-direction and magnitude of loading -strain rates -duration of the inter-stimulus interval -distribution and number of loading cycles per day	[46,47] [48] [49] [50]
**structure of bone**	-differences in mechanical properties between hard cortical bone (e.g. shafts of long bones) and elastic trabecular bone (e.g. hip bone necks and heads, vertebral bodies)	[28,32,40]
**muscle-bone interaction**	- mechanical stresses of contracting muscles on the skeleton - effect of myokines released by skeletal muscles on bone remodeling and regeneration	[45] [89]
**age and sex**	- bone sensitivity to mechanical stresses in relation to pubertal status - effect of estrogen signaling in osteocytes on inhibition of sclerostin expression - sexual dimorphism of irisin at rest and in response to exercise - aging-related decrease in sensitivity of the skeleton to mechanica loading due to: ✓decrease in angle between the neck and shaft of the femur ✓changes in osteocyte lacunocanalicular network ✓decrease in skeletal muscles size and function ✓changes in myokine production, e.g. the higher post-training concentration of irisin in younger versus older males, ✓impairment of loading-induced Wnt signaling and reduced osteoblast proliferative response ✓impairment of GH signaling (impaired mitochondrial function in osteocytes) ✓increased osteocyte apoptosis associated with estrogen deficiency	[70,98,125] [86] [91] [31] [108] [137] [138] [134] [79] [83]
**nutritional factors**	-effect of calcium intake on bone tissue adaptation to physical activity in children (an increase in leg BMC) -relationship of vitamin D and iron status to cortical bone health in physically active individuals -effect of vitamin D intake (both diet and supplements) on lower risk of developing and overload fracture -effect of carbohydrate provision on reducing the IL-6 response to exercise in healthy individuals	[98] [22] [120] [116]
**inflammatory factors**	-role of pro-inflammatory cytokines (TNFα and IL-1β) in inhibiting the up-regulation of NO production in osteocytes after mechanical stimulation	[20]

Based on the published studies of training programs directed on preventing bone fractures, when trying to recommend physical activity we should consider the different needs of patients, their body mass, age, sex, health status and also their level of physical fitness, capacity and previous training experience. Therefore, it is necessary to pay attention to individualize the type of activity and training loads (the intensity, duration, frequency). In their review, Kim et al. [157] suggested that a two-way communication approach between specialists and individuals is most effective in recommending exercises, among other things, for adequate exercise adherence. Moreover, exercises with greater loads/intensity than low-intensity walking, in adults and older people, previously inactive, should be preceded by cardiological tests [158] and an assessment of the condition of the skeletal system (primarily the occurrence of osteoporosis), especially in women in the peri- and post-menopausal period.

The published studies showed that high-impact (such as jumping) and resistance exercises are most effective in strengthening bone tissue. Therefore, this type of training is often recommended to prevent fractures, even in old age. However, depending on the stage of life, certain restrictions and precautions should be considered.

Exercises selected for children and adolescents should support the achievement of high peak bone mass, but on the other hand, training loads must not lead to overloading of the musculoskeletal system (so as not to inhibit the growth process). Teachers should be educated in this area. It is important to introduce osteogenic exercises during sport classes e.g. some jumping jacks, games outside. In adults, especially those working for many hours in a sitting position, changes of position are recommended. Increased muscle condition and impact on the skeleton can be achieved by counteracting the forces of gravity, for example by stair climbing [159]. This kind of activity may be more easily incorporated into working time or daily living than a specially organized activity. The participation in dance or step-aerobic classes may be the attractive form of osteogenic activity for women.

Proper physical activity can effectively inhibit or even reverse the decline of BMD in older people, even those with osteoporosis. However, when trying to recommend physical activity, especially in older people with little training experience, it is important to consider safety during exercise. Although high-intensity resistance training has been used and has been safe even for postmenopausal women with low or very low bone mass, it requires close supervision [75]. Elderly and obese people may be recommended for aerobic activity (for example, walking at a pace adapted to physical capacity) due to its metabolic effects, especially in terms of localizing adipocytes in bone marrow cavities [154]. Considering the health aspects of aerobic training programs and our previous experience in postmenopausal women, this type of physical activity with some modifications may be efficient to achieve osteogenic effects e.g. introducing some exercises with special equipment that increase the body contact area with the water during aqua aerobics [43] or poles with integrated resistance shock absorber during the Nordic walking program [44]. However, increasing the load during aerobic activity requires prior medical consultation. Exercises with a physiotherapist are also important for increasing balance and muscle strength in elderly people with low mobility. This is particularly important in preventing falls.

When trying to determine the amount of physical activity for effects on bone tissue, we can refer to the results of a study with the representative cohort of postmenopausal women (1681 participants) aged > 50 years from the National Health and Nutrition Examination Survey (NHANES) [160]. Authors calculated the metabolic equivalent (MET) hours per week (MET-hour/week) and demonstrated that high levels of physical activity (38 MET-hour/week) were superior to low levels of physical activity (11.9MET-hour/week) in improving total spine BMD in postmenopausal women. However, the subgroup analysis showed that the associations between BMD and the higher physical activity level were apparent in postmenopausal women aged<65 years or those with body mass index (BMI) <25 kg/m^2^. Elderly and obese people may require more individualized training loads.

Apart from the type of physical activity and exercise intensity, training frequency is a key aspect of successful training protocols. In a systematic review and meta-analysis (including seven studies with 17 exercise groups), Zitzmann et al. [161] determined the effect of training frequency on aBMD at the lumbar spine and hip (weight bearing/impact exercise or resistance exercise). The authors observed the significantly superior effect of higher, (i.e. two sessions and more per week) compared to lower training frequency (1 to <2 sessions/week) on the lumbar spine but not the hip BMD.

In a study of postmenopausal women with osteoporosis, Zitzmann et al. [161] showed that although exercise training (1 or 2 years of daily brisk walking and gymnastic training) led to a significant increase in BMD at the lumbar spine, the detraining reverted results toward a level that was not significantly different from the control group. Therefore, it is important to emphasize that systematic and continued physical activity is needed to maintain the bone mass gained through physical training program.

## 5. Conclusion

Bone response to exercise is a multidisciplinary problem, and both mechanical and biochemical stimuli affect the mechanotransduction mechanism. Systemic factors, related to the hormonal and energetic status, significantly determine the sensitivity of bone tissue to mechanical loads, therefore bone tissue adaptation to mechanical stimuli is sex-dependent and different in adolescents than in adults and in the elderly. In the elderly, adequate forms of physical activity are important in the prevention of falls and thus bone fractures. In spite of exercises that significantly load the skeleton are the most effective for the prevention of osteoporosis, endurance-type or combined activities may be also included during ageing or for people with visceral adiposity. Thus, when selecting exercises to improve bone health, it is important to take into account age, as well as metabolic and musculoskeletal system conditions. Therefore, it may be necessary to individualise the selection of exercises in terms of their efficiency and also safety. However, it should be emphasized that in order to maintain the bone mass gained through the physical training program, it is necessary to continue regular physical activity.
